# Complications following metal bar removal after Nuss repair are rare in a duocentric retrospective evaluation

**DOI:** 10.1007/s00383-022-05250-8

**Published:** 2022-09-22

**Authors:** Andreas C. Heydweiller, Tatjana T. König, S. Tolga Yavuz, Martin Schwind, Stephan Rohleder, Christina Oetzmann von Sochaczewski

**Affiliations:** 1 Sektion Kinderchirurgie, Klinik und Poliklinik für Allgemein-, Viszeral-, Thorax- und Gefäßchirurgie, Universitätsklinikum Bonn, Venusberg-Campus 1, 53127 Bonn, Germany; 2 Klinik und Poliklinik für Kinderchirurgie, Universitätsmedizin der Johannes-Gutenberg-Universität Mainz, Mainz, Germany; 3 Klinik und Poliklinik für Allgemeine Pädiatrie, Universitätsklinikum Bonn, Bonn, Germany

**Keywords:** Funnel chest, Pectus excavatum, Minimally invasive repair, Metal bar explantation, Complications, Clavien–Dindo classification

## Abstract

**Purpose:**

Minimally invasive pectus excavatum repair has gained widespread acceptance and its results and complications are well-described. However, there is a substantial debate on the risks and frequencies of complications following metal bar removal. We, therefore, aimed to analyse all complications that occurred during and after metal bar removal at our two paediatric surgical centres.

**Methods:**

Bar removal surgeries were identified via procedural codes and electronic records were reviewed using a pre-specified data extraction chart. Both intra- and postoperative complications were included and the latter scored according to Clavien–Dindo. We analysed the influence of the pre-specified potential predictors age, sex, and the number of implanted metal bars on the occurrence of complications using logistic regression.

**Results:**

We included 279 patients with a median age of 19 years (interquartile range 17–20 years). 15 patients experienced 17 complications. Of 11 postoperative complications, only an enlarging pleural effusion required a chest drain in local anaesthesia, resulting in a Claven-Dindo grade IIIa, whereas the remainder were classified as grade I. Neither age (adjusted odds ratio (aOR) 0.97, 95% confidence interval (CI) 0.84–1.13, *P* = 0.73), nor sex (aOR 0.88, 95% CI 0.19–4.07, *P* = 0.87) or the number of bars (aOR 0.64, 95% CI 0.15–2.71, *P* = 0.547) did influence the occurrence of complications.

**Conclusion:**

Complications following metal bar removal were scarce in our duocentric retrospective series and usually of minor relevance. However, to address the perceived paucity of data on the frequency and severity of complications following metal bar removal, further studies, including large database research is necessary.

## Introduction

The Nuss repair [1] has gained widespread acceptance in the correction of pectus excavatum in children and adults [2–5]. While the knowledge basis on success and complications following implantation [5–10] is broad and detailed, the evidence on complications during metal bar removal is scattered [11]. In a survey of members of the Chest Wall International Group, 82 of 112 surveyed surgeons had experienced at least one complication during bar removal procedures [11]. Among them were regularly pneumo- and occasionally hematothoraces, which made the survey authors conclude that serious complications are underreported in the published larger case series because the reported complications were rare and did largely not require any intervention [11]. Consequently, a systematic review identified only a small number of serious complications following bar removal [12]. Due to the bemoaned lack of evidence on complications following metal bar removal after a Nuss-OP, we retrospectively assessed all patients for complications that underwent this operation at our two centres.

## Methods

### Study participants

We retrospectively included all metal bar removals following Nuss repair that were conducted between the 1st January 2009 and the 31st December 2020 in the two participating paediatric surgical departments of Mainz and Bonn. Patients were identified via the procedural code for an implant removal following correction of a pectus excavatum (OPS-Code 5–349.5). None of the eligible patients were excluded.

### Surgical technique

Bar removals were performed under general anaesthesia. Patients were in a supine position with both arms abducted and positioned towards the right side of the table. We routinely re-open the implant scar and only excise it if it is considered cosmetically deficient. The end of the bar and the stabiliser were located and dissected free from surrounding tissue using diathermy. After freeing the holes of the stabiliser from adhesions, the wire used for fixation of the stabiliser was untwisted, cut in half, and removed in its two parts in one centre, while the stabiliser was just removed following straightening of its end in the other centre. In cases where we had used pectus bar locking screws, they were unscrewed at this stage of bar removal. Afterwards, the stabiliser was freed from the bar itself. This process was repeated on the left side in the exact same fashion if bilateral stabilisers were used. Following these steps, the right end of the bar was bent upwards to slightly straighten its end and the bar was then removed in a cautious hemicyclic movement directed slightly dorsal and away from the chest wall on the patient’s right side in one centre. In the other centre, the left end of the bar was completely straightened and the bar was removed in a similar fashion through the incision on the patient’s right-hand side. During movement inside the thorax, the bar was canted ventrally to avoid scratching along the chest wall and also protect lungs, heart, and internal thoracic arteries. We then closed the chest wall in layers starting with the chest wall muscles, because we routinely implant the bar sub-muscularly.

### Data collection

Data extraction with anonymization at the source was carried out by specifically trained chart abstractors using a pre-specified data extraction chart. Its suitability had been determined by a pilot investigation at one of the departments, and some, randomly chosen, results were cross-checked by another investigator to ensure data extraction quality as recommended elsewhere [13]. We collected information on age, sex, length of stay, operation time, and any complication that occurred either intra- or postoperatively, and any treatment initiated because of the complications. Postoperative complications were graded using the classification of Clavien-Dindo [14], whose applicability to minimally invasive pectus excavatum repair had been shown before [15].

### Statistical analysis

Medians were compared using Mood’s test and the association between complications and length of hospital stay was evaluated using point biserial correlation via the correlation-package (version 0.8.1)[16]. Following our pre-specified analysis plan, we assessed whether the occurrence of complications could be associated with age, sex or the number of implanted metal bars. This was done via logistic regression using R (version 3.5.3) with its standard stats4-package [17].

## Results

We included 279 patients, of which 239 (85.7%) were male. Median patient age at metal bar removal was 19 years (interquartile range 17–20 years), with a lower median age of 18 (interquartile range 16–20) in females than in males (*z* = 2.7765, *P* = 0.0055), whose median age was 19 (interquartile range 18–20). In our cohort, two patients, one male and one female, had their initial operation quite early at an age of 6 and 8 years respectively, both due to severe and asymmetric disease associated with Marfan syndrome. 227 (81.4%) patients had one bar removed, while 48 (17.2%) had two bars, and only 4 (1.4%) had three bars removed. Median length of stay was 2 days (range 0–12) in the whole cohort and length of stay was weakly correlated to the occurrence of complications (*ρ* = 0.19, 95% confidence interval 0.06–0.33, *P* = 0.006).

Complications could be found in 15 (5.4%) patients, comprising 13 (86.7%) males, who experienced 17 complications in total. Of them, 6 (35.3%) occurred intraoperatively and 11 (64.7%) postoperatively, while two patients had both intra- and postoperative complications. Intraoperative complications consisted of ossifications in two patients, which were so severe in one patient that they required the use of thoracoscopy and bilateral thoracic incisions to remove the metal bars safely. Both patients had their implants removed after a regular time of three years scheduled in our study sites. The patient in whom thoracoscopy was necessary suffered from chronic juvenile arthritis, while the other one had no chronic diseases besides a congenital cataract. In two patients, a small pneumothorax occurred intraoperatively, but did not require any intervention. In another two patients, tiny metal fragments remained in situ. Among the postoperative complications also were two small pneumothoraces that resolved without treatment. Two patients experienced an impaired wound healing, one of which occurred in a patient with severe ossifications that required thoracoscopy. Another two patients developed a seroma and one patient had a haematoma. A wound infection occurred in one patient, managed via local antiseptic therapy, and another had a keloid formation at the incision. Finally, another patient, who had an intraoperative pneumothorax, also had a pre-operative pleural effusion due to bar dislocation that increased in size postoperatively. This pleural effusion was drained via chest drain placement in local anaesthesia and resulting in IIIa-grading according to the Clavien-Dindo classification, whereas the remaining complications were managed conservatively and thus scored grade I according to the Clavien-Dindo classification. Two patients (0.7%) of the cohort, a 15 and a 27 years old male who had their bars implanted for three years, developed a mild and a moderate recurrence during follow-up, none of which had any complications during metal bar removal.

None of the pre-specified independent predictors age (adjusted odds ratio 0.97, 95% confidence interval: 0.84–1.13, *P* = 0.73), sex (adjusted odds ratio 0.88, 95% confidence interval: 0.19–4.07, *P* = 0.87), and the number of implanted metal bars (adjusted odds ratio 0.64, 95% confidence interval: 0.15–2.71, *P* = 0.547) had any influence on the occurrence of complications.

## Discussion

Despite being considered a medium-risk procedure [11], the assessment of complication rates following metal bar removal after pectus excavatum repair via the Nuss-procedure, evidence on complications is scarce and major complications are considered to be underreported by the authors of a survey of the Chest Wall International Group [11]. This notion might not be refuted by a recent systematic review of the literature including even case reports, but not grey literature [12], as the published literature may not be correct for underreporting. This could only be tackled by more data, which we provide in our report.

Its complication rate is similar to that of the literature: *Nyboe* and colleagues reported a complication rate of 2.4% (8/343) in a patient cohort with the same median age and sex distribution, but their complications were more severe with three hemo- and three of five pneumothoraces that required chest drain placement [18]. This report does not mention any minor complications, such as those largely observed in our cohort, for which it remains unclear if these did not occur or have not been assessed at all. Similarly, another study did not mention minor complications but had a relatively large complication rate of 17.5% (43/246) including serious intraoperative bleeding, pneumothoraces, and pleural effusions [19]. Again, it remains unclear, if minor complications had been assessed at all. Thus, a comparison of their results to ours is difficult beyond the statement that the most frequent complications during bar removal were minor wound complications.

In a very large and heterogeneous cohort of 1821 patients, including many children as the mean age of 9.13 years suggests, only 3.95% of patients experienced complications [20]. In this cohort, wound complications also were the most frequent ones, whereas severe complications such as severe bleeding requiring open surgery, hemo- and pneumothorax were scarce [20]. Their number was within the ranges that could be expected for the occurrence of at least one severe complication, based on the relationship case numbers and the occurrence of complications described in the survey among the Chest Wall International Group [11]. Of note, both reports [19, 20] were not considered by the survey authors in their discussion [11], despite them being published earlier and reporting major complications. The most recent report on complications during bar removal reported a complication rate of 2.7% in 436 patients, but, again, only major complications such as lung injuries, hemo- and pneumothoraces were reported, whereas minor complications were not mentioned at all [21]. This is not surprising, as the survey authors already noted [11], it is often the case that reports focus on complication rates during the Nuss repair, while complications during bar removal are only colorandi causa as it was in this case, too [21]. Another group investigated the potential effects of age and separated their cohort of 283 patients with a complication rate of 3.2% (9/283) into adults and adolescents, but found no difference between them [22]. A finding that remains inconclusive, because the overall event rate is quite low and the authors did employ univariate statistics comparing proportions, but not regression analysis as one would have expected to establish an association of a potentially relevant predictor of complications. Interestingly, this is the only report so far that also assessed minor complications, which were less frequent than the more severe pneumothorax [22].

We found a slight association between the occurrence of complications and the length of hospital stay, which was not noted in the only study [22] that assessed this parameter so far. The lack of assessment of this parameter in several other reports is not surprising, because in many other health systems, the length of hospital stay is just some hours [18, 23], the survey of the Chest Wall International Group reported a mean of seven hours [11], whereas in Germany there are certain systematic financial incentives that favour operations to be conducted on inpatients [24].

In a considerably large study of 1574 patients, the overall complication rate was 4.1%, but the majority of the patients experienced more severe complications like relevant bleeding and pneumothorax that required surgical intervention [23], while we did not encounter significant bleedings from the internal thoracic arteries in our cohort. They used the Clavien–Dindo classification, which was not used in any of the earlier reports, and described 34 severe complications of Clavien–Dindo class IIIa-V [23], while in our cohort only one patient experienced a Clavien–Dindo class IIIa complication. However, a direct comparison is difficult, because this large report used the Clavien–Dindo classification also for intraoperative complications [23], although it was never intended to be used this way: “The classification of 2004 was developed to record postoperative complications, […]” [25]. Consequently, we did not score intraoperative complications that occurred in our cohort according to Clavien–Dindo. We opted against the use of the recently described ClassIntra [26] classification because its validation just took place for visceral surgery [27]. Paediatric and thoracic surgery patients were underrepresented in the validation cohort with just 3% of the prospectively included 2520 patients [26]. We, therefore, felt that our relatively small retrospective cohort might not be suitable to establish the use of this classification system for intraoperative complications in these patient groups.

Aside this classification issue, *Media* and co-workers described an association between complications and age, both using mean ages and arbitrarily set age categories [23]. Again, this was based on univariate statistics using the comparison of means and proportions, but not a regression analysis as one would have expected. Moreover, categorisation of continuous variables should be avoided to reduce the effects of potential confounders, and if categorisation is employed, the number of categories should not be that small [28]. This is particularly relevant because there were some hints from preceding research that suggested that age might be a factor in the occurrence of complications during bar removal [19]. This was also true for the number of bars during bar removal [19, 23], that was thus included as a continuous predictor in our pre-specified analysis, too. However, the event rate of complications in our cohort was just too low to be able to calculate reliable results, due to the low statistical power and the potentially high risk of bias, as indicated in simulation studies [29].

The ossifications we encountered in two patients could not be traced back to the length of bar implantation as they had their bars implanted for the scheduled time. However, the patient with the much more severe ossifications, requiring thoracoscopy and bilateral incisions to free and remove the bar, was also diagnosed with chronic juvenile idiopathic arthritis. This is of relevance, as it had been associated with heterotopic ossifications following total hip replacement [30] and of the temporomandibular joint [31]. Thus, one might be tempted to speculate that this pre-existent disease might have played a role in the formation of such severe ossifications.

Recurrences following bar removal have been described before in both children and adults [32], which have been attributed to chest wall regression during growth in young children [33]. However, similar changes in the chest wall configuration were also observed in older children [34] and adults [35], so it remains unclear to what extent these changes might contribute towards a recurrence of the pectus excavatum. We assume that the duration of bar implantation in our patients was not a decisive factor, because its time exceeded those of the preceding reports [33–35]. Further exploration of potential explanatory factors was not suitable due to the low number of events in our cohort.

Besides the low event rate for complications, further limitations of our study are the retrospective nature, which inevitably introduces a relevant risk of bias, despite taking all necessary precautions such as pre-specified variables and data-extraction charts, and conduction of a pilot investigation by specifically trained chart abstractors [13]. Another issue might be that the duo-centric approach, avoiding the bias introduced by having only a single site and reducing the potentially limited external validity, could have resulted in patients coming from a different parent population, although we consider this aspect to be of lesser importance. On the contrary, the strict application of the well-established [14, 25], also in pectus excavatum repair [15], Clavien–Dindo classification is a strength of our study, as is the duo-centric assessment of complications following metal bar removal.

Taken together, we report the first non-monocentric assessment of complications following metal bar removal after minimally invasive pectus excavatum repair and found complications to be rare and of minor relevance for the clinical course based on the classification of Clavien-Dindo. Due to the low frequency of complications, large-scale database research is necessary to identify potentially predictive factors for complications.

## Data Availability

The data that support the findings of this study are available from the corresponding author upon reasonable request.
